# Cardiopulmonary Bypass during Cardiac Surgery Modulates Systemic Inflammation by Affecting Different Steps of the Leukocyte Recruitment Cascade

**DOI:** 10.1371/journal.pone.0045738

**Published:** 2012-09-19

**Authors:** Jan Rossaint, Christian Berger, Hugo Van Aken, Hans H. Scheld, Peter K. Zahn, Andreas Rukosujew, Alexander Zarbock

**Affiliations:** 1 Department of Anesthesiology, Intensive Care and Pain Medicine, University Hospital Münster, Münster, Germany; 2 Department of Anesthesiology, Intensive Care and Pain Medicine, University Hospital Münster, Münster, Germany; 3 Department of Thoracic and Cardiovascular Surgery, University Hospital Münster, Münster, Germany; 4 Department of Anesthesiology, Intensive Care Medicine, Palliative Care Medicine and Pain Management, University Hospital Bergmannsheil Bochum, Bochum, Germany; 5 Department of Thoracic and Cardiovascular Surgery, University Hospital Münster, Münster, Germany; 6 Department of Anesthesiology, Intensive Care and Pain Medicine, University Hospital Münster, Münster, Germany; Heart Center Munich, Germany

## Abstract

**Background:**

It is known that the use of a cardiopulmonary bypass (CPB) during cardiac surgery leads to leukocyte activation and may, among other causes, induce organ dysfunction due to increased leukocyte recruitment into different organs. Leukocyte extravasation occurs in a cascade-like fashion, including capturing, rolling, adhesion, and transmigration. However, the molecular mechanisms of increased leukocyte recruitment caused by CPB are not known. This clinical study was undertaken in order to investigate which steps of the leukocyte recruitment cascade are affected by the systemic inflammation during CPB.

**Methods:**

We investigated the effects of CPB on the different steps of the leukocyte recruitment cascade in whole blood from healthy volunteers (n = 9) and patients undergoing cardiac surgery with the use of cardiopulmonary bypass (n = 7) or in off-pump coronary artery bypass-technique (OPCAB, n = 9) by using flow chamber experiments, transmigration assays, and biochemical analysis.

**Results:**

CPB abrogated selectin-induced slow leukocyte rolling on E-selectin/ICAM-1 and P-selectin/ICAM-1. In contrast, chemokine-induced arrest and transmigration was significantly increased by CPB. Mechanistically, the abolishment of slow leukocyte rolling was due to disturbances in intracellular signaling with reduced phosphorylation of phospholipase C (PLC) γ2, Akt, and p38 MAP kinase. Furthermore, CPB induced an elevated transmigration which was caused by upregulation of Mac-1 on neutrophils.

**Conclusion:**

These data suggest that CPB abrogates selectin-mediated slow leukocyte rolling by disturbing intracellular signaling, but that the clinically observed increased leukocyte recruitment caused by CPB is due to increased chemokine-induced arrest and transmigration. A better understanding of the underlying molecular mechanisms causing systemic inflammation after CPB may aid in the development of new therapeutic approaches.

## Introduction

Cardiopulmonary bypass (CPB) triggers a systemic inflammation which is caused by an increase in the blood concentration of inflammatory markers and activation of immune cells [1], [2]. Organ dysfunction is attributed to a multitude of factors involving a cascade of inflammatory responses culminating in the inappropriate recruitment of leukocytes from the circulation [3]. Cellular indicators of inflammation include neutrophil activation and can be detected by measuring release of inflammatory cytokines as well as upregulation and release of adhesion molecules [4]. Increased blood cytokine concentrations and neutrophil activation are reported in acute complications after CPB [5]. Experimental and clinical studies implicate the activation of neutrophils in both acute and chronic vascular complications [6], [7], [8], [9].

Various treatment strategies have been tested to reduce the severity of the systemic inflammation induced by CPB and to improve the treatment, including anti-inflammatory drugs, novel components of the CPB, and new surgical techniques, but no single strategy has been proven effective yet [10], [11].

Leukocyte adhesion constitutes an essential process in the immune system, one that enables the accumulation of immune cells at sites of infection and inflammation [12]. Leukocyte recruitment occurs in a cascade of events that involves a series of adhesion and homing receptors [12]. In order to leave the vessel at sites of tissue damage or inflammation, leukocytes have to interact with and roll along the endothelium before they arrest and transmigrate into inflamed tissue [12]. Leukocyte capturing and rolling is mediated by selectins expressed on inflamed endothelial cells and their counter-receptors on neutrophils [13]. Slow rolling and arrest is predominantly mediated by integrins interacting with their ligands expressed on endothelial cells [14]. Integrins are members of a large family of functionally conserved adhesion receptors, which occur in low affinity conformational states on circulating leukocytes [15]. During rolling, leukocytes collect different inflammatory signals that activate intracellular signaling pathways [16]. Selectin engagement activates a signaling pathway in neutrophils causing the conformational change of integrins and the initiation of slow rolling [17]. This signaling pathway consists of the signaling molecules phospholipase (PLC) γ2, p38 mitogen-activated protein kinase (p38 MAPK), and Akt [18]. The binding of chemokines to their receptors induces the activation of signaling pathways in leukocytes which activates integrins (high affinity state) and induces leukocyte arrest [19]. Following arrest, integrins bound to their ligands transduce signals into leukocytes, which strengthen adhesion and induce transmigration.

The aim of this study was to elucidate how the CPB during cardiac surgery alters the different steps of the leukocyte recruitment cascade. By using flow chamber assays with human whole blood samples, *in vitro* transmigration assays, and biochemical phosphorylation experiments, we demonstrate that CPB during cardiac surgery abolishes selectin-mediated slow leukocyte rolling by altering intracellular signaling including the phosphorylation of PLCγ2, Akt, and p38 MAPK while increasing chemokine-induced arrest and transmigration of neutrophils.

## Materials and Methods

### Reagents

Unlike otherwise stated, all reagents were obtained from Sigma Aldrich (Taufkirchen, Germany).

### Observational study

To investigate the effects of CPB on the different steps of leukocyte recruitment, we conducted a prospective observational study in patients undergoing cardiac surgery for bypass grafting. This study was approved by the local ethic committee of the University of Münster (Institutional Review Board). Informed written consent for inclusion into the study was obtained from every patient more than 24 hours before enrollment. Patients received cardiac surgery for bypass grafting and were scheduled either for operation with the use of CPB (on-pump group) or surgery in off-pump coronary artery bypass-technique (OPCAB group). Two experienced, skilled cardiac surgeons were involved in this study and all on-pump and OPCAB patients were operated by these surgeons. The decision regarding the operation technique (on-pump or OPCAB) was only made by the surgeons based on the planned positions of the bypass grafts and overall patient status. 25 patients (7 on-pump, 9 OPCAB, 9 healthy volunteers) were included in this study and analyzed. The patients in both groups received the same anesthesia regiment with sodium thiopental, sufentanil, and cisatracurium for induction of anesthesia and sevoflurane or isoflurane to maintain general anesthesia. Blood samples were obtained after the induction of general anesthesia, immediately following protamine administration (on-pump and OPCAB), and 24 hours after the end of the surgical procedure. Healthy volunteers did not receive anesthesia or any type of surgery and donated whole blood samples at the corresponding time points. Patients were excluded from the study if they met one of the following exclusion criteria: age <18 years, pregnancy, immunosuppressive therapy within the last 7 days, chronic renal insufficiency (eGFR <60 ml/min), renal replacement therapy, pre-existing hematologic diseases and/or HIV-infection, acute of chronic inflammatory condition, organ transplantation or any malignancy in patient medical history.

### Blood-perfused human microflow chamber

To investigate selectin-mediated slow neutrophil rolling, we used a whole blood perfused human microflow chamber system as described previously [20], [21]. Briefly, glass capillaries were coated with E-selectin (3,5 µg/ml, R&D Systems, Minneapolis, MN, USA), P-selectin (20 µg/ml, R&D Systems, Minneapolis, MN, USA), E-selectin/ICAM-1 (3,5/3,5 µg/ml, R&D Systems, Minneapolis, MN, USA) or P-selectin/ICAM-1 (20/5 µg/ml, R&D Systems, Minneapolis, MN, USA) for 2 hours. Chambers were blocked with casein 1% (Fisher Scientific, Waltham, MA, USA) for 1 hour and afterwards perfused with heparinised whole blood samples at a constant shear stress of 5–6 dynes/cm^2^. It has been demonstrated before, that >90% of rolling cells in this system are polymorphonuclear neutrophils [20]. To investigate the chemokine-induced arrest of rolling neutrophils, a separate set of flow chambers was used. This flow chambers were coated with P-selectin (20 µg/ml), ICAM-1 (5 µg/ml) and Interleukin-8 (50 µg/ml, Peprotech, Rocky Hill, NJ, USA) or P-selectin and ICAM-1 as a control. Rolling and adhering cells per field of view were counted after 2 minutes of perfusion with whole blood at 5–6 dynes/cm^2^ and the ratio of adhering to rolling cells was calculated.

### *In vitro* transmigration assay

The transendothelial migration of isolated human neutrophils through a monolayer of cultured human umbilical vein endothelial cells (HUVEC) was performed as previously described [22]. Briefly, the 6.5 mm transwell filters (Corning Life Sciences, Corning, NY, USA) were coated with fibronectin (0.01%) for 1 hour. 4×10^4^ HUVECs were seeded per well and grown to confluence for 2 days. HUVECs were stimulated with TNF-α (5 nM, Peprotech, Rocky Hill, NJ, USA) for 16 hours. Human neutrophils were isolated from whole blood by Histopaque density centrifugation, resuspended in control plasma or plasma obtained from on-pump or OPCAB patients after administration of protamine and incubated at 37°C for 30 minutes in the presence or absence of a blocking antibody directed against Mac-1 (clone ICRF44, Biolegend, San Diego, CA, USA). 5×10^5^ neutrophils were applied on top of each transwell filter. Transmigration was allowed for 30 minutes and the number of transmigrated cells in the outer well was counted.

### FACS-analysis of neutrophils from human whole blood

The surface expression of leukocyte surface adhesion molecules on neutrophils was analyzed by flow cytometry. Human neutrophils were incubated with different antibodies (anti-human PSGL-1-antibody, clone KPL-1, BD Biosciences, Franklin Lakes, NJ, USA; anti-human LFA-1-antibody, clone MEM-25, ImmunoTools, Friesoythe, Germany; anti-human Mac-1-antibody, clone MEM-174, ImmunoTools) for 20 minutes. Samples were analyzed on a FACSCanto flow cytometer (BD Biosciences, Franklin Lakes, NJ, USA). FACS data was processed using FlowJo (version 7.5.5; Tree Star Inc., Ashland, OR, USA).

### Western blotting

For biochemical assays, isolated neutrophils from human whole blood were stimulated with immobilized E-selectin as described previously [23], [24]. Following stimulation, cells were lysed using RIPA buffer [23]. Lysate was boiled with Laemmli sample buffer at 95°C for 10 minutes, run on a 10% PAGE-SDS gel and immunoblotted with antibodies against Akt, phospho-Akt, PLCγ2, phospho-PLCγ2, p38 MAPK and phospho-p38 MAPK (all from Cell Signaling Technology, Danvers, MA, USA). Blots were developed using the Amersham ECL Prime detection system (GE Healthcare, Piscataway, NJ, USA).

### Statistical analysis

Statistical analysis was performed with SPSS (version 20.0) using one-way ANOVA, Student-Newman-Keuls test, post-hoc correction or t-test where appropriate. All data are represented as means ± SEM. A p-value <0.05 was taken as statistically significant.

## Results

### Selectin-mediated slow leukocyte rolling is abolished in patients after CPB

One of the first steps of the leukocyte recruitment cascade is the selectin-mediated transition from rolling to slow rolling [12]. Binding of selectins to their ligands on neutrophils induces the activation of an intracellular signaling pathway leading to activation of the β_2_-integrin LFA-1 which mediates slow leukocyte rolling [25]. In order to investigate the effect of cardiopulmonary bypass (CPB) on neutrophil slow rolling, we conducted a prospective observational study in patients undergoing cardiac surgery. There were no significant differences regarding demographic data, including age, weight and serum electrolytes between the patient groups (Table 1). There were no differences with respect to the clinical severity of the underlying cardiovascular disease, as detected by preoperative EuroSCORE, acute or previous myocardial infarctions, ejection fraction, presence of cerebrovascular diseases, and history of cerebral stroke (Table 1). We also included the NYHA and ASA scores.

**Table 1:** Demographic and clinical patient data.

	On-pump patients	OPCAB patients	p-value
**No. of patients**	7	9	
**Gender (m/f)**	6/1	6/3	
**Age [yrs]**	71±4	67±3	n.s.
**Body weight [kg]**	85.8±5.7	84.6±6.3	n.s.
**Body surface area [m^2^]**	2.05±0.08	1.99±0.09	n.s.
**CPB time [min]**	95±6	-	
**Aortic-clamp time** **[min]**	62±7	-	
**Procedure duration** **[min]**	230±20	181±61	n.s.
**Preoperative NYHA** **score**	2.0±0.26	2.38±0.18	n.s.
**ASA score**	2.83±0.31	2.88±0.23	n.s.
**Cardiovascular diseases**			
Previous MI (>90d)	2/7	4/9	n.s.
Acute MI (<90d)	2/7	4/9	n.s.
Ejection fraction [%]	54±7	50±3	
Cerebrovascular disease	1/7	2/9	
Stroke	0/7	2/9	
EuroSCORE	3.67 ±1.02	4.25±1.01	
**No. of treated vessels**	2.8±0.20	2.0±0.26	n.s.
**ICU days**	8±5	5±2	n.s.
**Transfused blood products** **[ml]**	219±88	292±77	n.s.

Values are presented as mean +/− SEM or numbers of patients. MI: myocardial infarction. ICU: intensive care unit. n.s.: not significant.

**Figure 1:**
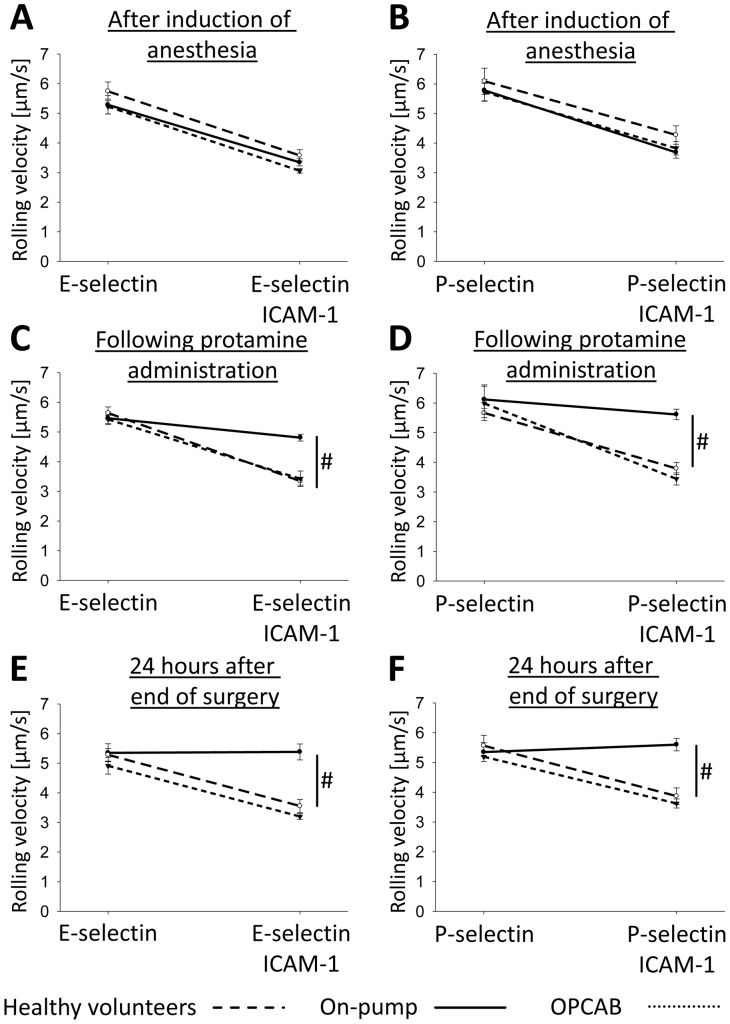
Selectin-mediated slow leukocyte rolling is abolished in patients after CPB. The rolling velocity of neutrophils in whole blood from healthy control volunteers (dashed line), on-pump patients (straight line) and OPCAB patients (dotted line) was measured after induction of anesthesia (A, B), following administration of protamine (C, D), and 24 hours after the end of surgery (E, F) using microflow chambers coated with E-selectin or P-selectin alone and in combination with ICAM-1. The average rolling velocity of neutrophils is presented as mean ± SEM (n = 7–9). The wall shear stress in all flow chamber experiments was 5–6 dynes/cm^2^. ^#^ p<0.05.

To investigate the effect of CPB on selectin-mediated slow leukocyte rolling, we used a previously published flow chamber system [20], [21]. The advantage of the flow chamber system is that the neutrophil rolling behavior can be investigated in whole blood without isolating neutrophils. This is important, because it has been shown that the isolation process might alter the activation status and rolling ability of neutrophils [26], [27], [28]. After the induction of anesthesia, neutrophils from both patient groups and healthy volunteers showed the same rolling velocity on E-selectin or P-selectin alone and a reduction of the rolling velocity on E-selectin or P-selectin in the presence of the LFA-1 ligand ICAM-1 (Figure 1 A+B). After the administration of protamine, neutrophils from on-pump patients failed to reduce their rolling velocity on E- or P-selectin when ICAM-1 was added to the substrate, whereas neutrophils from OPCAB patients and healthy volunteers showed a reduction of the rolling velocity on E-selectin and ICAM-1 or P-selectin and ICAM-1 (Figure 1 C+D). During cardiac surgery with the use of CPB and in OPCAB technique, systemic anticoagulation with heparin was antagonized with protamine. Onpump patients were anticoagulated before connection to the cardio-pulmonary bypass with heparin at a dose of 400 I.U./kg bodyweight. OPCAB patients received heparin at a dose of 200 I.U./kg bodyweight. Heparin was antagonized with protamine at a ratio of 1 I.U. protamine per 1 I.U. heparin. Onpump patients received 34583±2770 I.U. protamine, whereas OPCAB patients received 16375±1849 I.U. protamine (p = 0.00). In an additional experiment we investigated whether protamine can directly influence selectin-mediated slow leukocyte rolling. In order to show that protamine has no direct effect on neutrophil rolling and adhesion, whole blood samples from some healthy volunteers were treated with protamine at a dose of 0, 16 or 32 I.U./ml, corresponding to the administration of protamine at 0, 200 or 400 I.U./kg bodyweight in patients (as calculated from the assumption of 80 ml blood per 1 kg bodyweight). No difference in neutrophil rolling and arrest was observed between the groups (data not shown).

Interestingly, slow neutrophil rolling in on-pump patients was still defective 24 hours after the end of the surgical procedure (Figure 1 E+F). These data suggest that the use of CPB during cardiac surgery abolishes selectin-mediated integrin activation and subsequently alters neutrophil recruitment.

### CPB during cardiac surgery increases chemokine-induced arrest of neutrophils

**Figure 2:**
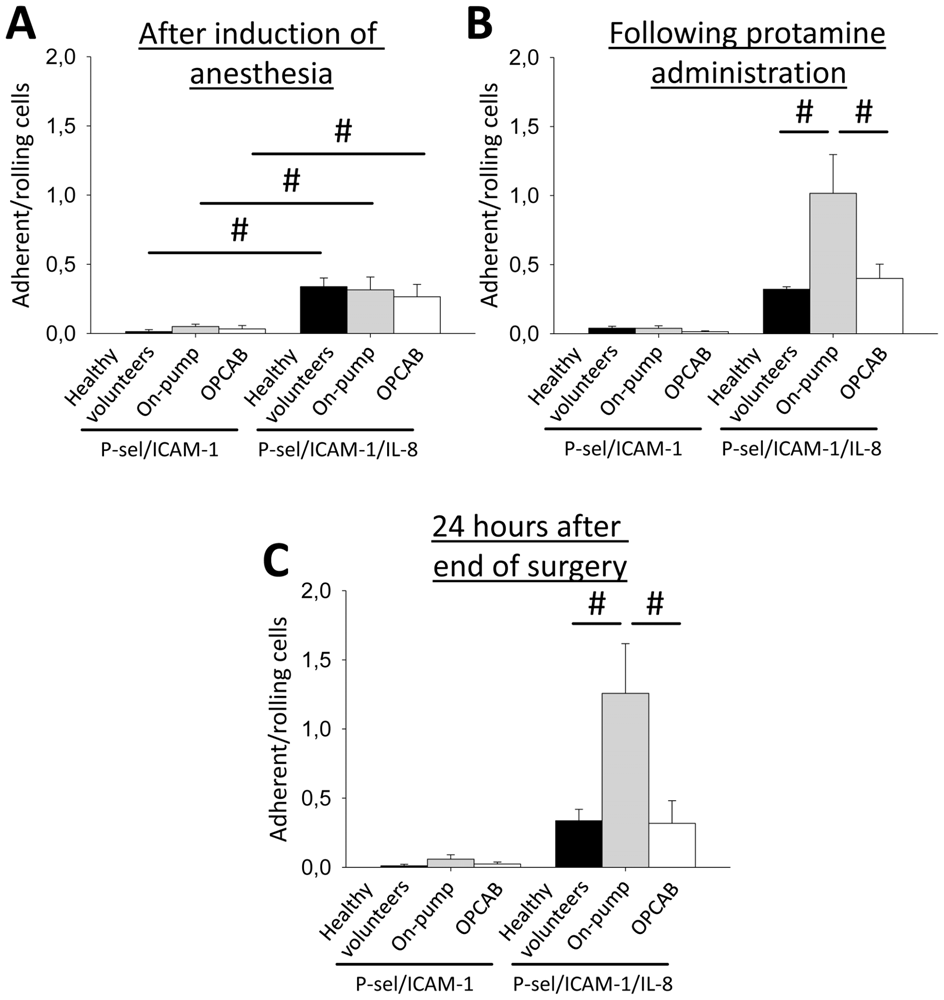
CPB during cardiac surgery increases chemokine-induced leukocyte arrest. Chemokine-induced arrest of neutrophils was investigated in a human microflow chamber assay. Flow chambers coated with P-selectin/ICAM-1 or P-selectin/ICAM-1/Interleukin-8 were perfused with whole blood from healthy control volunteers (black bars) or from cardiac surgery patients operated with the use of CPB (on-pump patients, grey bars) or from patients operated in OPCAB technique (OPCAB patients, white bars) after induction of anesthesia (A), after the administration of protamine (B), and 24 hours after the end of the surgery (C). The ratio of adherent/rolling cells was calculated after 2 minutes of perfusion at a constant wall shear stress of 5–6 dynes/cm^2^ (n = 7–9). ^#^ p<0.05.

During rolling along the endothelium, neutrophils are activated by different chemokines presented by inflamed endothelial cells [12]. Binding of chemokines to their receptors on neutrophils induces integrins activation and leads to leukocyte arrest [19]. Similar to selectin-mediated slow leukocyte rolling, chemokine-induced neutrophil arrest is LFA-1 dependent [29]. This process occurs partly overlapping with selectin-mediated slow rolling. To investigate the effect of CPB on chemokine-induced arrest, we performed experiments with flow chambers coated with P-selectin, ICAM-1 and immobilized interleukin (IL)-8. After inducing anesthesia, the ratio of adherent neutrophils to rolling neutrophils in flow chambers coated with P-selectin and ICAM-1 was low in both patient groups and in healthy volunteers at the corresponding time point (Figure 2A). The ratio of adherent neutrophils to rolling neutrophils in flow chambers coated with P-selectin, ICAM-1, and IL-8 significantly increased in all three groups (Figure 2A). After administration of protamine, the ratio of adherent to rolling neutrophils was increased in on-pump patients compared to OPCAB patients and controls at the corresponding time point (Figure 2B). This effect was still present 24 hours after the end of the surgical procedure (Figure 2C). These data show that the use of CPB increases chemokine-induced arrest of circulating neutrophils.

### CPB does not alter the expression of adhesion molecules on neutrophils involved in slow leukocyte rolling and chemokine-induced arrest, but increases Mac-1 expression

**Figure 3:**
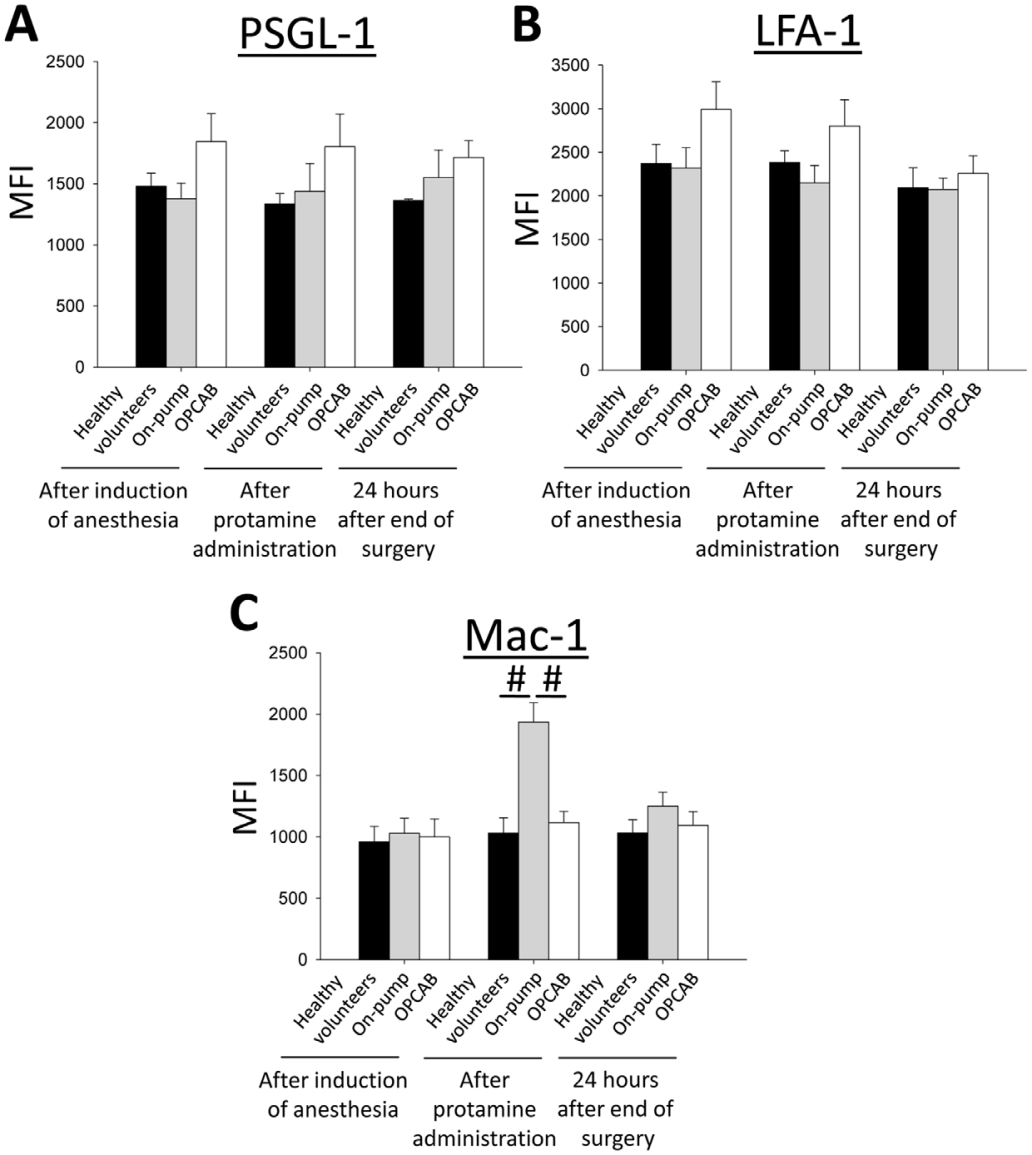
CPB does not alter the expression of adhesion molecules on neutrophils involved in slow leukocyte rolling and chemokine-induced arrest, but increases Mac-1 expression. The surface expression of different adhesion molecules on human neutrophils from healthy control volunteers (black bars), patients undergoing cardiac surgery with CPB (on-pump patients, grey bars) or without CPB (OPCAB patients, white bars) were analyzed by flow cytometry after induction of anesthesia, after administration of protamine, and 24 hours after the end of the surgery. The mean fluorescence intensity as a measure for surface expression of PSGL-1 (A), LFA-1 (B), and Mac-1 (C) was quantified (n = 7–9). ^#^ p<0.05.

Activation of neutrophils might be associated with the change of the surface expression of different adhesion molecules including PSGL-1 and integrins [30], [31], [32]. To elucidate whether the effects of CPB on neutrophil slow rolling and chemokine-induced arrest is caused by an altered expression profile of surface adhesion molecules involved in leukocyte recruitment, we investigated the surface expression of PSGL-1, LFA-1, and Mac-1 on neutrophils by flow cytometry. The expression of PSGL-1 (Figure 3A) and LFA-1 (Figure 3B) on neutrophils from both patient groups and from healthy volunteers was not significantly different after inducing anesthesia, following protamine administration, and 24 hours after the end of the surgical procedure. In contrast, the expression level of Mac-1 on neutrophils from on-pump patients was significantly elevated after the end of CPB compared to the other groups (Figure 3C). However, the increased Mac-1 expression on neutrophils from on-pump patients cannot explain the altered selectin-mediated slow leukocyte rolling and chemokine-induced arrest, because both steps are dependent on the β_2_-integrin LFA-1 [29], [33]. In order to show that Mac-1 is not involved in slow leukocyte rolling and chemokine-induced arrest, we blocked Mac-1 in whole blood samples obtained from healthy volunteers with a monoclonal antibody and showed that the blockade of Mac-1 has no effect on selectin-mediated slow rolling and chemokine-induced adhesion (data not shown).

### CPB affects intracellular signaling

**Figure 4:**
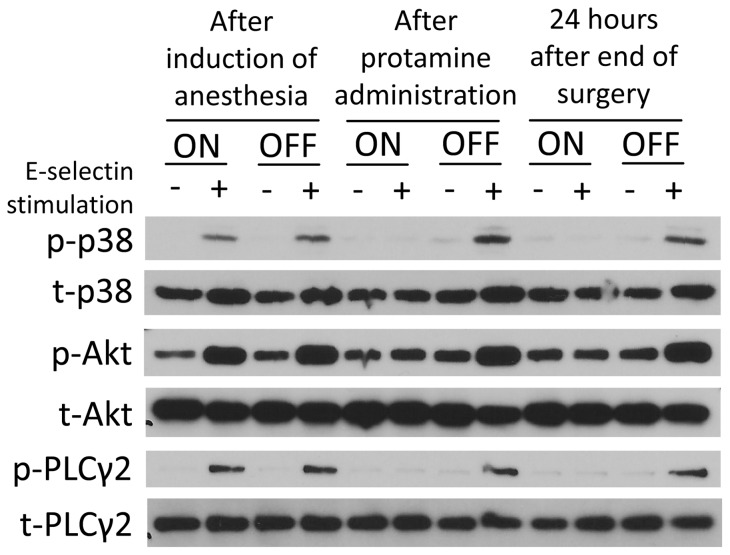
CPB affects intracellular signaling. The phosphorylation of PLCγ2, Akt, and p38 MAPK was investigated by western blot. Neutrophils derived from patients undergoing cardiac surgery with CPB (ON) or without CPB (OFF) obtained after induction of anesthesia, after administration of protamine, and 24 hours after the end of the surgery were left unstimulated or stimulated by rolling on E-selectin–coated wells for 10 minutes. Lysates were prepared and immunoblotted with antibodies against phosphorylated PLCγ2 (phospho PLCγ2 (Tyr1217)), total PLCγ2, phosphorylated Akt (phospho Akt (S473)), total Akt, phosphorylated p38 MAPK (phospho-p38), or total p38 MAPK (exemplary blot of 3 experiments).

It is known that binding of selectins to their counter ligands on neutrophils induces the activation of an intracellular signaling cascade leading to integrin activation and slow leukocyte rolling *in vivo* [13], [34]. Following selectin engagement, Btk is phosphorylated in a Syk-dependent manner, and the signaling pathway downstream of Btk divides into PLCγ2- and PI3Kγ-dependent branches, which both regulate β_2_-integrin–mediated slow rolling [35]. p38 MAPK is located downstream of PLCγ2 [24], [36]. To study, whether the observed effects of CPB on neutrophil rolling velocity might be caused by a disruption of intracellular signal transduction, we investigated the phosphorylation of PLCγ2, Akt (as a readout for PI3K activation), and p38 MAPK. Phosphorylation of PLCγ2, Akt, and p38 MAPK after E-selectin stimulation could be detected in neutrophils from OPCAB patients and on-pump patients after inducing anesthesia (Figure 4). However, immediately after the administration of protamine, E-selectin-induced phosphorylation of PLCγ2, Akt, and p38 MAPK was completely abolished in neutrophils from on-pump patients, whereas E-selectin induced phosphorylation of these molecules was normal in neutrophils from OPCAB patients (Figure 4). The abolished phosphorylation of PLCγ2, Akt and p38 MAPK following stimulation in neutrophils from on-pump patients was still present 24 hours after the end of the surgical procedure (Figure 4). These data suggest that CPB inhibits selectin-mediated integrin activation by interfering with intracellular signaling pathways.

### CPB causes increased Mac-1 dependent neutrophil transmigration *in vitro*

**Figure 5:**
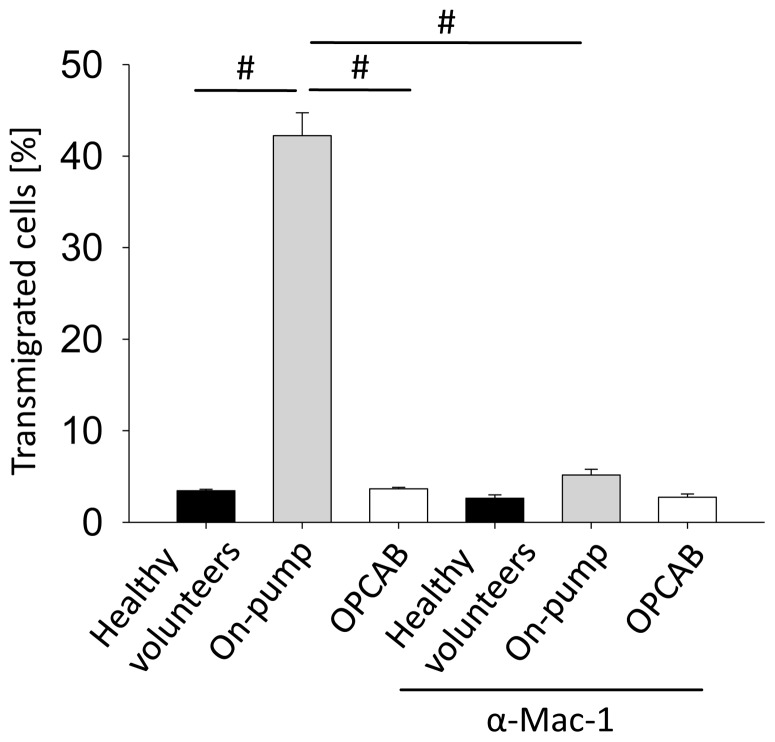
CPB causes increased Mac-1 dependent neutrophil transmigration *in vitro.* Isolated neutrophils from healthy volunteers were preincubated with control serum (black bars) or serum obtained from cardiac surgery patients operated with the use of CPB (grey bars) or in OPCAB technique (white bars) at 37°C for 30 minutes in the presence or absence of a blocking Mac-1 antibody. The neutrophils were allowed to transmigrate through a confluent HUVEC monolayer in a transwell system *in vitro*. The endothelial cell layer has been stimulated with TNF-α for 16 hours. After 30 minutes, the number of transmigrated neutrophils was determined (n = 3). ^#^ p<0.05.

The last step in the leukocyte recruitment cascade is transmigration, when the leukocytes leave the vessel and emigrate into the inflamed tissue [12]. To investigate the effect of CPB on the transmigration process, we performed an *in vitro* transmigration assay using a transwell system with TNF-α-stimulated human umbilical vein endothelial cells (HUVEC) and isolated neutrophils from healthy volunteers incubated with plasma from on-pump patients, OPCAB patients, or healthy volunteers. Neutrophils incubated with plasma from healthy volunteers and OPCAB patients (obtained after protamine administration) show similar transmigration rates through the TNF-α-stimulated HUVEC-monolayer (Figure 5). However, the number of transmigrated neutrophils increased after 30 minutes of incubation with plasma obtained from on-pump patients (obtained after protamine administration, Figure 5). This effect could be nearly completely reversed by pre-incubation with a blocking Mac-1 antibody (Figure 5), suggesting that CPB increases the Mac-1 dependent transmigration by a direct effect on neutrophils.

## Discussion

Lamy and colleagues have shown that the overall 30-day mortality is not different between cardiac surgery patients with off-pump or on-pump coronary-artery bypass grafting [37]. However, this study and other studies demonstrated a significantly lower incidence of acute kidney injury, respiratory complications, and postoperative delirium in the off-pump groups compared to on-pump patients. These postoperative complications have multifactorial causes, including hypoperfusion, hypoxia and inflammation. The CPB circuit induces a release of pro-inflammatory cytokines and activation of leukocytes resulting in systemic inflammation [2], [3]. The inflammatory response and the inappropriate displacement of leukocytes frequently induces organ dysfunction after using a CPB circuit [38]. These organ dysfunctions may contribute to postoperative complications and procedure-associated mortality [1], [39], [40]. Although cardiac surgery without CPB reduces the inflammatory response [41], it does not modulate hypoperfusion and hypoxia during the procedure. This goes along with our results, showing that cardiac surgery with CPB is associated with increased leukocyte recruitment. As the pathogenesis of the organ dysfunction is multifactorial, it is not surprising that modulating inflammation does not translate into a survival benefit. However, reducing inflammation and additional causes promoting organ dysfunction (e.g. hypoxia and hypoperfusion) may reduce mortality under these conditions.

The mechanisms by which CPB alters leukocyte recruitment remain unknown. A better understanding of the molecular pathways causing systemic inflammation might be valuable for the improvement of the clinical management of patients with cardiac surgery. By performing a clinical trial, we here show that CPB alters different steps of the leukocyte recruitment cascade. The use of a CPB circuit during cardiac surgery abolished selectin-mediated slow leukocyte rolling. This effect appears to be mediated by altered intracellular signaling rather than by changing the surface expression of adhesion molecules. However, chemokine-induced arrest and transmigration was significantly increased after cardiac surgery with CPB, showing that the different steps of the recruitment cascade are differently affected by the use of a CPB circuit.

During CPB-induced inflammation, selectins expressed on inflamed endothelial cells mediate the first contact between leukocytes and endothelial cells and initiate leukocyte recruitment. The subsequent activation of neutrophils by a number of pro-inflammatory mediators, including platelet-activating factor (PAF) and IL-8, provokes an increase of CD11b/CD18 (Mac-1) integrin levels on the leukocyte surface [42]. Released cytokines and chemokines upregulate the expression of intercellular adhesion molecule (ICAM)-1, vascular cell adhesion molecule (VCAM)-1, and platelet-endothelial cell adhesion molecule (PECAM)-1 on the surface of endothelial cells [43]. The binding of integrins to ICAM-1 and VCAM-1 initiates firm adhesion of leukocytes to endothelial cells, leading to their transendothelial migration into the tissue. Here, leukocytes release their lysosomal contents [44], [45]. These agents stimulate lipid peroxidation of endothelial cells and myocyte membranes, causing cellular dysfunction, edema and cell death [46]. CPB is associated with increased levels of soluble adhesion molecules [47]. Higher levels of adhesion molecules are briefly expressed and return to normal within a few hours but they are believed to be responsible for the dysfunction of multiple organ systems observed in the postoperative period [47]. A relationship between adhesion molecule expression and inflammatory mediators has also been shown [48].

To further study the underlying mechanisms of the altered leukocyte recruitment during cardiac surgery with CPB, we used a newly developed microfluidic whole blood perfusion system originally developed for mouse blood [25], [49]. The advantage of this system is that neutrophils can be investigated in whole blood without isolation. This is important, because it has been shown that the isolation process might alter the activation status and rolling ability of neutrophils.

We studied the rolling of neutrophils from on-pump patients, OPCAP patients, and healthy volunteers in the autoperfused flow chamber assay. Neutrophils from all patients demonstrated similar rolling velocities on P-selectin and E-selectin. Neutrophils from on-pump patients, however, did not display reduced rolling velocities on P-selectin/ICAM-1 or E-selectin/ICAM-1 after protamine administration. These data suggest that the signaling pathway linking PSGL-1 to integrin activation is disturbed. It has been demonstrated that this pathway is important for Gα_i_-independent leukocyte recruitment [18], [23], [25], [50].

Leukocyte adhesion molecules, including selectins and their ligands, play a crucial role in leukocyte recruitment [51]. Nonetheless, we could not detect changes in surface expression of adhesion molecules involved in selectin-mediated integrin activation on neutrophils (PSGL-1 and LFA-1).

Since CPB drastically affects selectin-dependent leukocyte rolling, we next investigated the intracellular, selectin-dependent signaling pathways. Both the PI3Kγ- and PLCγ2-dependent pathways and their phosphorylation are crucial for leukocyte recruitment [18]. Whereas we could find E-selectin-stimulated phosphorylation in neutrophils from OPCAP patients, we could not detect E-selectin-stimulated phosphorylation in neutrophils from on-pump patients. We therefore hypothesize that CPB inhibits selectin-mediated integrin activation by means of interference with intracellular signaling pathways and thereby ultimately impairs slow leukocyte rolling. Similar to these findings, we could recently show that acute uremia also abolishes selectin-mediated slow leukocyte rolling [21]. However, the molecular mechanisms of disturbed selectin-induced intracellular signaling remain elusive.

However, in contrast to other adhesion molecules, Mac-1 (CD11b) was up-regulated on neutrophils from on-pump patients. Neutrophil CD11b expression is up-regulated during systemic inflammation caused by different stimuli [52], [53], [54]. and positively correlates with indicators of disease severity [55] and adverse outcomes [56], [57]. Our data are in accordance to the findings of another study showing that the surface expression of CD11b is up-regulated following cardiac surgery with CPB [42]. However, increased CD11b expression does not necessarily equate to augmented CD11b-dependent adhesion [58], [59] which is requisite for neutrophil-endothelial interactions [60]. Increased expression of conformationally active CD11b is seen on circulating leukocytes during CPB [61] and systemic inflammation [57], [62], [63]. Based on these data and the fact that neutrophil transmigration is Mac-1 dependent, [64] our observation of an increased transmigration of neutrophils incubated with plasma from on-pump patients can be explained. Blocking Mac-1 by a blocking antibody completely inhibited neutrophil transmigration in the transwell assay, showing that the increased transmigration of neutrophils incubated with plasma from on-pump patients is Mac-1 dependent. The increased chemokine-induced arrest and transmigration may be implicated in inadvertent tissue injury during cardiac surgery with CPB. The reasons why anti-Mac-1 strategies failed to improve outcomes in human trails may be a result of the failure of antibodies to specifically inhibit conformationally active CD11b on circulating leukocytes [65]. The effectiveness of blocking GPIIa/IIIb in acute coronary syndromes may be partially attributed to its ability to bind conformationally active CD11b and inhibit Mac-1 mediated leukocyte adhesion [66].

It is known that hypoxia, which may occur during cardiac surgery, can cause an inflammatory response by signaling pathways involving the hypoxia-inducible transcription factor (HIF) [67]. It has been demonstrated that HIF-1α, a key sensor of ischemia-reperfusion, is up-regulated in cardiac surgery patients during CPB and is associated with increased release of inflammatory mediators [68]. However, the precise molecular signaling pathways leading to Mac-1 up-regulation on neutrophils during CPB are still unknown. A possible mechanism for this observations is that the different proinflammatory mediators released during CPB directly activate neutrophils [69] and induce the up-regulation of Mac-1 on the cell surface. Another possible mechanism is that the exposition of blood to non-physiological surfaces during CPB activates platelets [70] which subsequently may interact with neutrophils and form platelet-neutrophil aggregates [71]. It is known that this interaction induces the up-regulation of Mac-1 on neutrophils [72].

The expression of HIF-1α during hypoxic conditions has been shown to trigger the production and release of adenine nucleotides [67], [73]. These mediators cause an increased expression of Mac-1 on the cell surface of neutrophils, similar to our observations during CPB [74]. In addition, HIF-1α enhances the endothelial production of TNF-α by activation of NF-kB [67], [75], which may cause the enhanced recruitment of neutrophils into different organs in response to CPB. TNF-α can also directly activate neutrophils and up-regulate Mac-1 on the cell surface [76].

Our study has some limitations. Although the transfused blood products did not significantly differ between the groups, it has to be considered that blood transfusion may have an influence on inflammation [77]. In addition, the low number of patients included in this study does not entirely rule out the possibility of additional factors affecting leukocyte activation. The regulation of proteins involved in circadian rhythmicity has recently been shown to play a role in the control of HIF (hypoxia-inducible factor)-dependent cardiac metabolism and ischemia tolerance [78]. However, since all patients underwent surgery in the morning and experiments with blood from healthy volunteers were conducted at the same time point, we can exclude a possible effect of circadian rhythmicity on the observed results.

To this end, our data show that CPB during cardiac surgery impairs leukocyte slow rolling, but significantly elevates chemokine-induced arrest and transmigration. The defect in selectin-mediated integrin activation appears to be, at least partially, due to decreased phosphorylation of selectin-dependent intracellular signaling. The increased transmigration is caused by an increased Mac-1 expression on circulating neutrophils after CPB. These data suggest that the inappropriate leukocyte displacement during CPB-induced inflammation is caused by an increased chemokine-induced arrest and transmigration.

The pharmacological blockade of Mac-1 could be a promising target for a novel therapeutic approach to reduce leukocyte recruitment during CPB-induced systemic inflammation and dampen the severity of this condition in cardiac surgery patients.
